# ﻿Phylogeny and comparative analysis of mitochondrial genomes of *Gomphus* spp. Pers. (Basidiomycota, Agaricomycetes), with descriptions of *G. matijun* J.W. Liu & F.Q. Yu and *G. bijiensis* sp. nov.

**DOI:** 10.3897/mycokeys.124.158670

**Published:** 2025-11-13

**Authors:** Xianyi Wang, Zhongyao Guo, Jiawei Tao, Yunhan Guo, Guoyu Wang, Guangyin Xu, Qirui Li, Hongmei Liu

**Affiliations:** 1 Engineering Research Center of Health Medicine, biotechnology of Institution of higher education of Guizhou Province, Guizhou Medical University, Guiyang, China Guizhou Medical University Guiyang China; 2 Engineering Research Center of Medical Biotechnology, School of Biology and Engineering, Guizhou Medical University, Guiyang, China Laboratory Animal Center of Guizhou Medical University Guiyang China; 3 Laboratory Animal Center of Guizhou Medical University, Guiyang, China Guizhou Medical University Guiyang China; 4 The High Efficacy Application of Natural Medicinal Resources Engineering Center of Guizhou Province (The Key Laboratory of Optimal Utilization of Natural Medicine Resources), School of Pharmaceutical Sciences, Guizhou Medical University, Guiyang, China Laboratory Animal Center of Guizhou Medical University Guiyang China

**Keywords:** Clavarioid fungi, comparative genomics, evolutionary adaptation, fungal diversity, Gomphaceae

## Abstract

The genus *Gomphus* Pers. presents persistent taxonomic challenges due to its morphological similarities with related genera. In this study, we collected two specimens of *Gomphus* from Guizhou, China; one specimen is described as a new species, *Gomphus bijiensis***sp. nov.** and the other is identified as *G. matijun* J.W. Liu & F.Q. Yu based on morphological traits and phylogenetic analyses of the nuclear rDNA internal transcribed spacer (ITS) and nuclear rDNA large subunit (LSU). To resolve their evolutionary relationships we assembled and annotated the mitochondrial genomes of both species using next-generation sequencing. Comparative analyses revealed codon usage strongly biased toward A- or U-ending codons, consistent with the low GC content typical of fungal mitochondria. Variation in protein-coding gene lengths and base composition suggests that diverse evolutionary pressures have shaped these genomes. Divergence time estimation indicates that morphological diversity within *Gomphus* and related macrofungi has largely resulted from convergent evolution. Phylogenetic reconstruction places *G. bijiensis* and *G. matijun* within a distinct clade, supporting their close evolutionary affinity and the coexistence of ancestral and derived traits. This study provides the first comprehensive mitochondrial genomic data for *Gomphus*, offering new insights into its taxonomy, phylogeny, and evolutionary dynamics, and establishing a framework for future studies within the Gomphaceae.

## ﻿Introduction

The genus *Gomphus* Pers. (family Gomphaceae) represents a taxonomically challenging group due to its pronounced morphological overlap with related genera. First established by Persoon in 1797 with *G. clavatus* designated as the type species, the genus has since undergone repeated reassessment. Morphologically, *Gomphus* species are typically fan-shaped to funnel-shaped, display distinctive coloration, and form ectomycorrhizal associations with members of the Fagaceae, Myrtaceae, and Pinaceae (Giachini 2004). This variation in macromorphology has frequently obscured taxonomic boundaries, contributing to the reassignment of several species into allied genera such as *Gloeocantharellus* Singer, *Phaeoclavulina* (Corner) Brinkmann, and *Turbinellus* Earle (Giachini et al. 2010). Molecular phylogenetic analyses further complicate the picture, with earlier studies suggesting paraphyly within the Gomphaceae (Miller 2002). On the basis of Giachini’s taxonomic framework and subsequent molecular evidence, *Gomphus* sensu stricto, once comprising about 35 species, has now been reduced to 16 accepted species (Index Fungorum, accessed August 2025). *Gomphus* is primarily distributed in subtropical and temperate regions of Asia, Europe, and North America. Within the PR of China, eight species are recognized, including *G. clavatus*, *G. roseus* R.H. Petersen, *G. matijun*, and the newly described taxon presented in this study (Liu et al. 2022). This update highlights the increasing recognition of *Gomphus* diversity, particularly in Asia, where ongoing molecular and morphological studies continue to refine its taxonomy (Singh et al. 2024).

The morphological variability within the genus includes traits such as the wrinkled to nearly poroid structure of the hymenium and the warty (verrucose) nature of basidiospores, once considered distinctive to *Gomphus*, which have now been observed in other genera within Gomphaceae (Humpert et al. 2001). This morphological convergence has caused confusion and debate among mycologists, complicating the accurate identification and systematic placement of *Gomphus* species (Scambler et al. 2018).

In addition to morphological complexities, the ecological roles of *Gomphus* species underscore their importance in forest ecosystems. As ectomycorrhizal fungi, they form symbiotic relationships with key forest trees, facilitating the exchange of nutrients and promoting the growth and health of their hosts (Fan et al. 2023). This ecological significance requires an understanding of *Gomphus* taxonomy to inform conservation efforts and protect forest biodiversity (Ye et al. 2020).

Molecular studies have further highlighted the complexity of *Gomphus* classification. Earlier molecular phylogenetic analyses revealed that *Gomphus* sensu lato—which includes genera such as *Gloeocantharellus* Singer, *Phaeoclavulina* (Corner) Brinkmann, and *Turbinellus* Earle, as well as *Ramaria* sensu lato—are paraphyletic within the Gomphaceae (Giachini et al. 2011). These findings demonstrate the complex evolutionary relationships within the family and the need for taxonomic refinement. In response, several taxonomic rearrangements have occurred over the past few decades (Liu et al. 2022), resulting in the reclassification of some species previously placed in *Gomphus* into other genera (Petersen 1973; Villegas et al. 1999).

Nouhra highlighted that several species within *Gomphus* sensu lato lacked clear diagnostic traits, creating persistent uncertainty regarding their taxonomic placement (Nouhra et al. 2005). Building on these earlier observations, Giachini and Castellano (2011) further refined the genus by demonstrating that *Gomphus* sensu stricto can be distinguished by specific features, including violet to lavender-brown or milky coffee-colored hymenia, recurrent clamp connections, and verrucose spores. These refinements helped clarify the boundaries of *Gomphus* s. str. within the Gomphaceae, although some taxonomic ambiguities remain.

In recent years, the study of mitochondrial genomes (mitogenomes) have gained attention as a powerful tool for phylogenetic analyses. The mitogenome, maternally inherited and relatively small, offers a valuable source of genetic information for reconstructing evolutionary relationships among species (Sun et al. 2022). Its high mutation rate and frequent recombination make it particularly useful for distinguishing closely related species and tracing their evolutionary history (Luo et al. 2024). This genetic approach has enabled researchers to uncover evolutionary lineages with greater precision, allowing for the identification of species that were previously overlooked due to morphological similarities (Li et al. 2023). Moreover, mitogenome analysis provides insights into the timing of evolutionary events, allowing researchers to estimate species divergence times and construct more accurate phylogenetic trees. This method is especially useful in cases where fossil records are sparse or incomplete, offering a genetic perspective on the evolutionary history of organisms (Arcones et al.2021).

The increasing availability of mitogenome data, combined with advanced computational tools, has revolutionized our understanding of fungal evolution and diversification. As more mitogenomes are sequenced, new insights into the processes shaping species boundaries and evolutionary trajectories are expected. In this study, we investigate the mitogenomes of two *Gomphus* species collected from Guizhou, China, including one previously undescribed taxon. Alongside comparative mitogenomic analyses, we formally identify and described this new species, *G. bijiensis* sp. nov., integrating morphological and molecular evidence. The objectives of our study are: (1) to formally describe *G. bijiensis* as a distinct lineage within the genus; (2) to elucidate the structural and compositional features of the two *Gomphus* mitogenomes; (3) to identify variation and conservativeness between *Gomphus* and other Gomphaceae mitogenomes through comparative analyses; (4) to determine the phylogenetic position of *Gomphus* within the Basidiomycota using mitochondrial gene datasets; and (5) to examine the evolutionary significance of *Gomphus* morphology through divergence-time estimation. This work represents the first publication of complete mitogenomes in *Gomphus*, while simultaneously advancing its taxonomy and deepening our understanding of the evolutionary dynamics of the Gomphaceae.

## ﻿Methods

### ﻿Specimen collection and preservation

Specimens were collected and catalogued from distinct locations in Guizhou Province, PR of China (Rathnayaka et al. 2025). The specimen of *G. matijun* was collected from the Qiannan Buyi and Miao Autonomous Prefecture, while *G. bijiensis* was collected from Niubang Township in Weining County, Bijie City. Following collection, specimens were identified morphologically and their habitats were photographed. The samples were then air-dried naturally and deposited at the Engineering Research Center of Medical Biotechnology, Guizhou Medical University, with specimen identifiers MY1010 and MY449.

### ﻿Morphological identification

To identify the species, detailed morphological examinations were conducted on both fresh and dried specimens. Macroscopic features, such as overall shape, size, coloration, surface texture, and hymenophore arrangement, were described using the terminology outlined in Giachini (Giachini et al. 2012). Macroscopic features were examined and photographed using the Nikon SMZ-745T stereomicroscope (Nikon, Japan). Spore ornamentation was further analyzed using the Phenom LE scanning electron microscope (Phenom, Netherlands). Measurements were based on at least 30 spores per specimen, excluding abnormally shaped or immature spores. Morphological descriptions were compared with taxonomic treatments and regional keys in Flora Fungorum Sinicorum (Liu et al. 2022) and other relevant monographs to ensure accurate identification and comparison with previously described *Gomphus* taxa.

### ﻿DNA extraction

Genomic DNA from two *Gomphus* species were extracted using the DNeasy Plant Mini Kit (Qiagen, Germany) according to the manufacturer’s protocols, with modifications to optimize DNA yield and purity for downstream genomic analysis. Freshly collected or herbarium-preserved specimens were used for DNA extraction. To minimize contamination and ensure DNA quality, small pieces of inner context tissue were carefully excised from the fruiting bodies. For fresh collections, tissue was taken immediately after field sampling while for preserved material dried specimens deposited in the herbarium were used. Uncontaminated internal tissue was selected to obtain reliable molecular data. Approximately 50 mg of tissue was excised from the pileus (cap) region of the fruiting bodies, ensuring a representative sample for DNA extraction.

The tissue samples were initially ground into a fine powder using a sterile mortar and pestle under liquid nitrogen to break down the cell walls and facilitate the release of cellular contents. The powdered tissue was then transferred into a 1.5 mL microcentrifuge tube, to which 400 µL of the provided lysis buffer (AP1) was added. The samples were incubated at 65 °C for 10 minutes, allowing for cell lysis. During this time, proteins, lipids, and other cellular materials were solubilized, making the DNA more accessible for extraction.

Following incubation, the samples were briefly centrifuged to remove any large particulate matter, and an equal volume of buffer AP2 was added to neutralize the lysate. The mixture was then vortexed and centrifuged at 13,000 rpm for 5 minutes to separate the debris from the supernatant, which contained the genomic DNA.

The supernatant was transferred to a new tube, and the DNA was precipitated by adding an equal volume of 100% ethanol. After mixing, the sample was centrifuged at 13,000 rpm for 10 minutes to pellet the DNA. The DNA pellet was washed twice with 70% ethanol to remove any residual impurities. Following air drying, the DNA pellet was resuspended in 50 µL of sterile, deionized water.

The quality and concentration of the extracted DNA were assessed using a NanoDrop spectrophotometer (Thermo Fisher Scientific) by measuring the absorbance at 260 nm (A260) and 280 nm (A280). The purity of the DNA was evaluated by calculating the A260/A280 ratio, which typically ranged from 1.8 to 2.0, indicating high-quality DNA suitable for downstream applications such as PCR amplification, sequencing, and genome assembly.

For long-term storage, the extracted DNA was kept at -20 °C for future analysis. The final DNA samples were ready for PCR amplification, sequencing, and other molecular analyses aimed at identifying species-specific markers and constructing the complete mitogenomes of the two *Gomphus* species.

### ﻿Molecular identification

Molecular identification of the novel *Gomphus* species was performed using DNA barcoding techniques. PCR was conducted using universal primers for the nuclear ribosomal internal transcribed spacer (ITS) region and the large subunit rDNA (LSU) gene (Xue et al. 2019), both of which are used in species identification. PCR reactions were performed in a 25 µL volume containing extracted DNA, primers, dNTPs, Taq DNA polymerase, and buffer. The thermocycler protocol consisted of an initial denaturation step followed by cycles of denaturation, annealing, and extension, culminating in a final extension step.

The internal transcribed spacer (ITS) and nuclear large subunit (nLSU) regions were amplified using primer pairs ITS1/ITS4 and LR0R/LR5, respectively (Xue et al. 2019). PCR reactions were performed in 25 μL volumes containing 1 × reaction buffer, 2.5 mM MgCl_2_, 0.2 mM dNTPs, 0.4 μM of each primer, 1 μL Taq DNA polymerase (Takara, Dalian, PR of China), and approximately 30 ng of template DNA. The thermocycler program consisted of an initial denaturation at 94 °C for 4 min, followed by 35 cycles of denaturation at 94 °C for 40 s, annealing at 55 °C for 45 s, extension at 72 °C for 1 min, and a final extension at 72 °C for 10 min. PCR products were purified using a Gel Extraction Kit (Omega Bio-Tek, USA) and sequenced bi-directionally at Sangon Biotech (Shanghai).

The resulting sequences were edited and assembled using Geneious Prime 2023.2.1 software (https://www.geneious.com). Consensus sequences were compared against reference sequences in the NCBI GenBank (Suppl. material 1: table S1) database via BLAST to confirm species identity. Phylogenetic analyses were performed using PhyloSuite v1.2.3 (Zhang et al. 2020), an integrated platform for phylogenetic reconstruction that incorporates maximum likelihood (ML) methods. For ML analysis, the optimal evolutionary model was selected using ModelFinder within PhyloSuite, and the tree was constructed with IQ-TREE (1,000 bootstrap replicates) to assess branch support.

### ﻿Mitochondrial genome sequencing, assembly, and annotation

The mitogenomes were sequenced using next-generation sequencing technology. Raw sequence data were assembled using Geneious Prime 2023.2.1 (Wang et al. 2025a) and aligned with the mitochondrial reference sequence of *Turbinellus floccosus* (Schwein.) Giachini, Osmundson, A. Sánchez-García & Spatafora (NC045218) and *Ramaria flavescens* (Schaeff.) Quél. (PP847337). Genome annotation was conducted using MFannot (Valach et al. 2014) and MITOS (Bernt et al. 2013) based on the mitochondrial genetic code 4 to identify protein-coding genes (PCGs), open reading frames (ORFs), rRNAs, tRNAs, and introns in the two *Gomphus* mitogenomes. ORFs exceeding 100 amino acids were further analyzed through BLASTP searches against the NCBI Open Reading Frame Finder and the NCBI non-redundant protein sequence database. The annotated tRNA genes were validated using tRNAscan-SE v1.3.1 software (Chan et al. 2021), and physical maps of the two *Gomphus* mitogenomes were constructed using OGDraw v1.2 (Greiner et al. 2019).

### ﻿Base composition and strand asymmetry analysis

The base composition of the mitogenomes of *Gomphus* and other Gomphaceae species were determined using DNASTAR Lasergene v7.1 (http://www.dnastar.com/). Strand asymmetry was calculated using the formulas: GC skew = [G − C] / [G + C] and AT skew = [A − T] / [A + T].

### ﻿Codon usage and substitution rate analysis

Codon usage in the *Gomphus* mitogenomes were analyzed using Sequence Manipulation Suite (Stothard 2000) with genetic code 4. The nonsynonymous (Ka) and synonymous (Ks) substitution rates of protein-coding genes (PCGs) in the mitogenomes of *Gomphus* and other Phallomycetidae were calculated using DnaSP v6.10.01 (Rozas et al. 2017).

### ﻿Genetic distance and genome homology

The genetic distance between each pair of the 15 major PCGs was determined using the Kimura-2 parameter (K2P) substitution model in MEGA v6.06. Genome-wide homologies among the seven chimeric species were assessed using AliTV (https://alitvteam.github.io/AliTV/d3/AliTV.html), with all mitochondrial genomes aligned starting from the *cox1* gene.

### ﻿Phylogenetic analysis

A comprehensive molecular approach was used to construct a phylogenetic tree of Basidiomycetes based on mitochondrial genes. Two datasets were created from 59 Basidiomycetes: (1) PCG, consisting of the concatenated sequences of 15 conserved PCGs, and (2) PCG12, comprising the first and second codon positions of these 15 conserved PCGs. *Tremella fuciformis* Berk. (Ascomycota) was used as the outgroup. Individual mitochondrial genes were aligned using MAFFT v7.037. The aligned genes were then concatenated into a single mitochondrial gene set using PhyloSuite v1.2.3 (Zhang et al. 2020). A preliminary partition homogeneity test was conducted to assess phylogenetic inconsistencies among genes. The best-fit evolutionary model and partitioning scheme were determined using PartitionFinder 2.1.1 (Lanfear et al. 2017). Phylogenetic trees were constructed using Bayesian inference (BI) with MrBayes v3.2.6 (Ronquist et al. 2012) and maximum likelihood (ML) with IQ-tree v1.6.3 (Nguyen et al. 2015).

### ﻿Phylogenetic analysis and time-divergence estimation

A time-divergence phylogenetic tree was constructed using 59 mitogenomes. The complete mitogenomes were aligned using the MAFFT v7.037 software to ensure accurate alignment of homologous regions. Poorly aligned regions were identified and removed using Gblocks v0.91b to enhance the reliability of the alignment.

Bayesian inference (BI) analyses were performed using MrBayes v3.2.7 (Ronquist et al. 2012) with two independent runs, each comprising four chains, for 10 million generations, sampling every 1,000 generations. The average standard deviation of split frequencies fell below 0.01, indicating convergence between runs. The first 25% of sampled trees (2,500 trees) were discarded as burn-in, and the remaining trees were used to construct a majority-rule consensus tree. Posterior probabilities were calculated from the post-burn-in trees to assess branch support. Convergence and effective sample size (ESS > 200) were further confirmed using Tracer v1.7 (Drummond et al. 2012).

For time-divergence estimation, a molecular clock model was implemented using BEAST v2.6.3. The alignment was analyzed under an uncorrelated log-normal relaxed clock model, which accounts for rate variation among lineages. Calibration points, based on fossil records and previously published divergence times, were applied to estimate the divergence times of key nodes (Wang et al. 2025b). The Markov chain Monte Carlo (MCMC) analysis was run for a sufficient number of generations, with parameters sampled at regular intervals.

The resulting phylogenetic tree, annotated with divergence times, was initially visualized using FigTree v1.4.4 (http://tree.bio.ed.ac.uk/software/figtree/). For further refinement of the visual presentation, we utilized Adobe Illustrator to optimize the tree’s graphical elements. Referencing the taxonomic hierarchy and divergence time frameworks from TimeTree (http://timetree.org/), adjustments were made to enhance clarity, including refining branch lines and standardizing node labeling.

## ﻿Results

### ﻿Identification of a new *Gomphus* species

To confirm the novelty of the new species, BLAST analyses of its ITS and nLSU sequences were performed against the NCBI nucleotide database (nr/nt) using BLASTn. For the ITS sequence, the top hit was *G. clavatus* (PQ652817), with 85.78% identity, 94% query coverage, an E-value of 0, and a max score of 891. The second closest match was *G. ludovicianus* R.H. Petersen & Justice, N. Amer (NR169660) with 85.19% identity, 91% coverage, an E-value of 0, and a max score of 873. For the nLSU sequence, the highest similarity was observed with *G. matijun* (NG228916) (96.03% identity, 91% coverage, E-value=0, max score=1539), followed by *G. ludovicianus* (NG241961) (95.51% identity, 97% coverage, E-value=0, max score=1,519). The relatively low sequence identities (<98%) with known species in both ITS and nLSU regions further support the recognition of this taxon as a new species.

**Figure 1.**
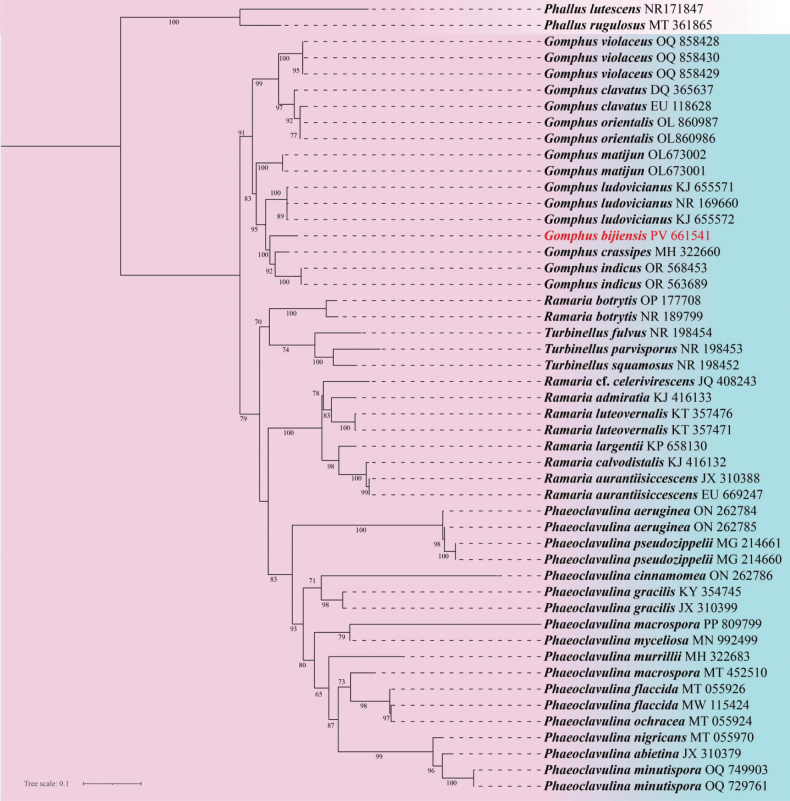
Maximum likelihood (ML) phylogeny of 49 fungal species based on ITS sequences. Bootstrap support values (≥65%) are indicated at the nodes. The newly described species *G. bijiensis* is highlighted in red, while *G. matijun* and other ingroup taxa are shown in black. Outgroup taxa are indicated with a lighter background.

The maximum likelihood (ML) phylogenetic tree inferred from ITS sequences (Fig. 1) provides strong molecular evidence in support of recognizing *Gomphus bijiensis* as a distinct species. In this tree, *G. bijiensis* forms a well-supported monophyletic clade with a bootstrap value of 100, clearly separated from other congeners. In addition, the phylogenetic tree inferred from LSU sequences (Suppl. material 2) revealed that *G. bijiensis* was distinctly separated from other congeneric species within *Gomphus*, which is congruent with the ITS-based inference in reflecting its affinity to these taxa while still maintaining genetic distinctiveness. Collectively, both molecular datasets strongly support the designation of *G. bijiensis* as a phylogenetically independent species within *Gomphus*.

### ﻿Taxonomy

#### ﻿ Gomphus bijiensis

Taxon classificationFungiGomphalesGomphaceae

Wang, Guo & Liu sp. nov.

D5E8CDE3-C127-56CC-82A3-71FEBF01F562

Fungal Names: FN 572719

Fig. 2a–c

##### Diagnosis.

*Gomphus bijiensis* is phylogenetically distinct from *G. indicus* K. Das, Hembrom & R. Kujur and *G. matijun* (bootstrap support = 100) and differs morphologically in having (1) smaller basidiocarps (6–11 cm vs. up to 15 cm in *G. indicus*), (2) a darker violet to purplish-brown pileus (6F6–7E8 vs. lighter brownish-orange in *G. matijun*), and (3) smaller, more prominently warted basidiospores [(7.5–)8.0–9.8(–10.5) × (4.5–)5.0–6.0(–6.5) μm vs. larger and less ornamented spores in *G. indicus*].

##### Etymology.

The epithet *bijiensis* refers to the type location, Guizhou Province, PR of China.

##### Holotype.

**PR of China** • Guizhou Province, Bijie City, collected on soil under a *Pinus* forest, August 2023, coll. MY1010 (Herbarium of Guizhou Medical University).

##### Description.

Basidiocarps are robust, funnel-shaped to irregularly lobed, pileus 6–11 cm diam., violet to purplish-brown (Munsell: 6F6–7E8), surface rugulose to wrinkled. Hymenophore with blunt, forked folds, lacking lamellae, concolorous with pileus but paler at maturity. Stipe 4–9 × 1.5–2.5 cm, solid, tapering toward base, surface whitish to cream (4A2–4A3) with brown fibrils. Flesh white, unchanging. Basidiospores (7.5–)8.0–9.8(–10.5) × (4.5–)5.0–6.0(–6.5) μm, Q = 1.45–1.72, Qm = 1.58 ± 0.06 (n = 50), ellipsoid to broadly ellipsoid, hyaline, thick-walled, with irregular warted ornamentation. Basidia 35–42 × 7–9 μm, clavate, 4-spored, with basal clamp connections. Hyphae in trama hyaline, 3–6 μm wide, with irregularly thickened walls, clamp connections present.

**Figure 2.**
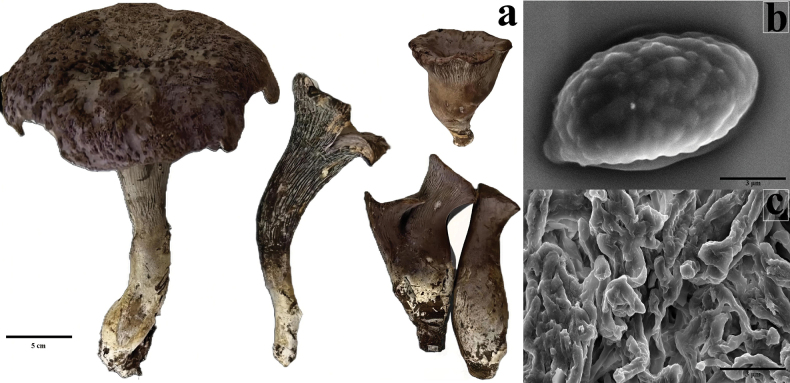
*Gomphus bijiensis*, a. Fresh samples; b. SEM images of basidiospores showing warted ornamentation; c. SEM images of mycelial structure.

##### Habit, habitat, and distribution.

Growing gregariously on soil in mixed *Pinus* forests. Known only from the type locality in Bijie City, Guizhou Province, PR of China (approx. 27°05'10"N, 103°48'50"E).

##### Notes.

*Gomphus bijiensis* is phylogenetically closely related to *G. indicus* and *G. matijun*, but both molecular and morphological data support its recognition as a distinct species. It differs from *G. indicus* in its smaller basidiocarps, darker pileus coloration, and more prominently ornamented spores, while it differs from *G. matijun* in pileus colour (deep violet to purplish-brown vs. brownish-orange), narrower stipe, and smaller spore size. The combination of these diagnostic traits, along with strong bootstrap support in phylogenetic analysis, justifies recognition of *G. bijiensis* as a novel species.

#### ﻿ Gomphus matijun

Taxon classificationFungiGomphalesGomphaceae

J.W. Liu & F.Q. Yu, in Liu, Luangharn, Wan, Wang & Yu, Mycoscience 63 (6): 294 (2022)

3D224910-4B39-55BF-8D58-B8A019E5D49C

841963

Fig. 3a–c

##### Notes.

**Figure 3.**
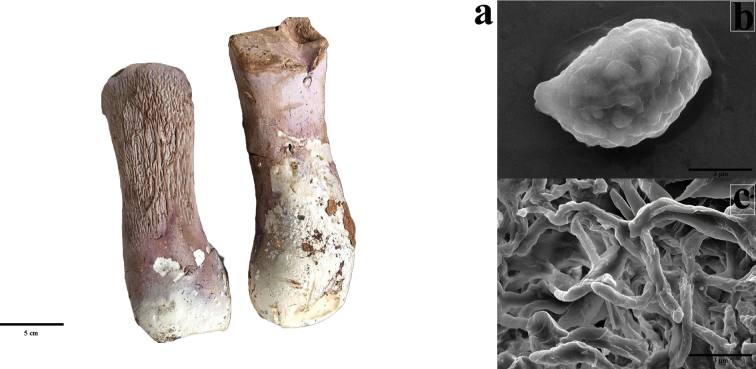
*Gomphus matijun*, a. Fresh samples; b. SEM images of basidiospores showing warted ornamentation; c. SEM images of mycelial structure.

*Gomphus matijun* was recently described from Guizhou Province, southern PR of China (Liu et al. 2022). This species is characterized by unipileate fruiting bodies at maturity, ellipsoidal to elongate basidiospores measuring 9–11 × 6–7 μm, and predominantly 2-spored basidia. In this study, we included *G. matijun* as a comparative taxon to evaluate the phylogenetic placement of *Gomphus* using mitochondrial genome data. We carefully examined both fresh and dried specimens, confirming their identity by comparison of ITS and LSU sequences with published data. A photo plate of fresh samples, SEM images of basidiospores showing warted ornamentation, and SEM images of the mycelial structure are given (Fig. 3). For the complete morphological description, readers are referred to the original publication (Liu et al. 2022).

### ﻿Modified key to *Gomphus* species

**Table:** 

1a	Basidiomata dividing into multiple lobes at maturity, pileus fan-shaped	**2**
1b	Basidiomata single-pileate at maturity, pileus funnel-shaped to flattened, rarely fan-shaped	**3**
2a	Pileocystidia present; pileus orange-brown to dark violet; basidiospores 10–15 × 4–7.5 µm; Asia, Europe, North America	***G. clavatus***
2b	Pileocystidia absent	**3a**
3a	Basidiospores less than 13 µm long	**4**
3b	Basidiospores 13 µm or longer	**6**
4a	Basidiospores 7.5–10 × 3.5–5 µm; pileus brown to rosaceous; hymenophore irregular, reticulate to nearly poroid; Africa (Cameroon, DRC, Uganda)	***G. brunneus***
4b	Basidiospores 7.5–11 µm long, otherwise	**5**
5a	Basidiospores 7.5–10 × 4.5–6 µm; pileus violet to purplish-brown; hymenophore shallowly wrinkled, without true lamellae; Guizhou, China	***G. bijiensis***
5b	Basidiospores 9–11 × 6–7 µm; pileus grayish-blue to blue-purple; hymenophore wrinkled, concolorous or gray; southeastern China	***G. matijun***
6a	Basidiospores 13–15 × 5.5–6 µm; pileus yellow-brown to orange-brown; hymenophore violet; Algeria, Morocco, Spain	***G. crassipes***
6b	Basidiospores variable in length and width, not matching above	**7**
7a	Basidiospores 11.5–15 × 6–9 µm; pileus bluish-violet; China	***G. violaceus***
7b	Basidiospores 10.3–15.5 × 4.3–7.5 µm; pileus yellowish-brown to purplish-brown; SW China	***G. orientalis***
7c	Basidiospores exceeding 15 µm	**8**
8a	Basidiospores 15–21.5 × 6–7.5 µm; pileus pale yellow to ash-blonde; hymenophore light to greyish-violet; India	***G. indicus***
8b	Basidiospores 14–17 × 5–7 µm; pileus dull brown to drab, bruising darker; hymenophore finely wrinkled, purple-gray; southeastern USA	***G. ludovicianus***

The following dichotomous key is based on morphological characteristics and includes all currently accepted *Gomphus* species worldwide (sensu Giachini et al. 2010, and subsequent molecular studies), with reference to the latest research (Singh 2024).

### ﻿Characterization of the two *Gomphus* mitochondrial genomes

**Figure 4.**
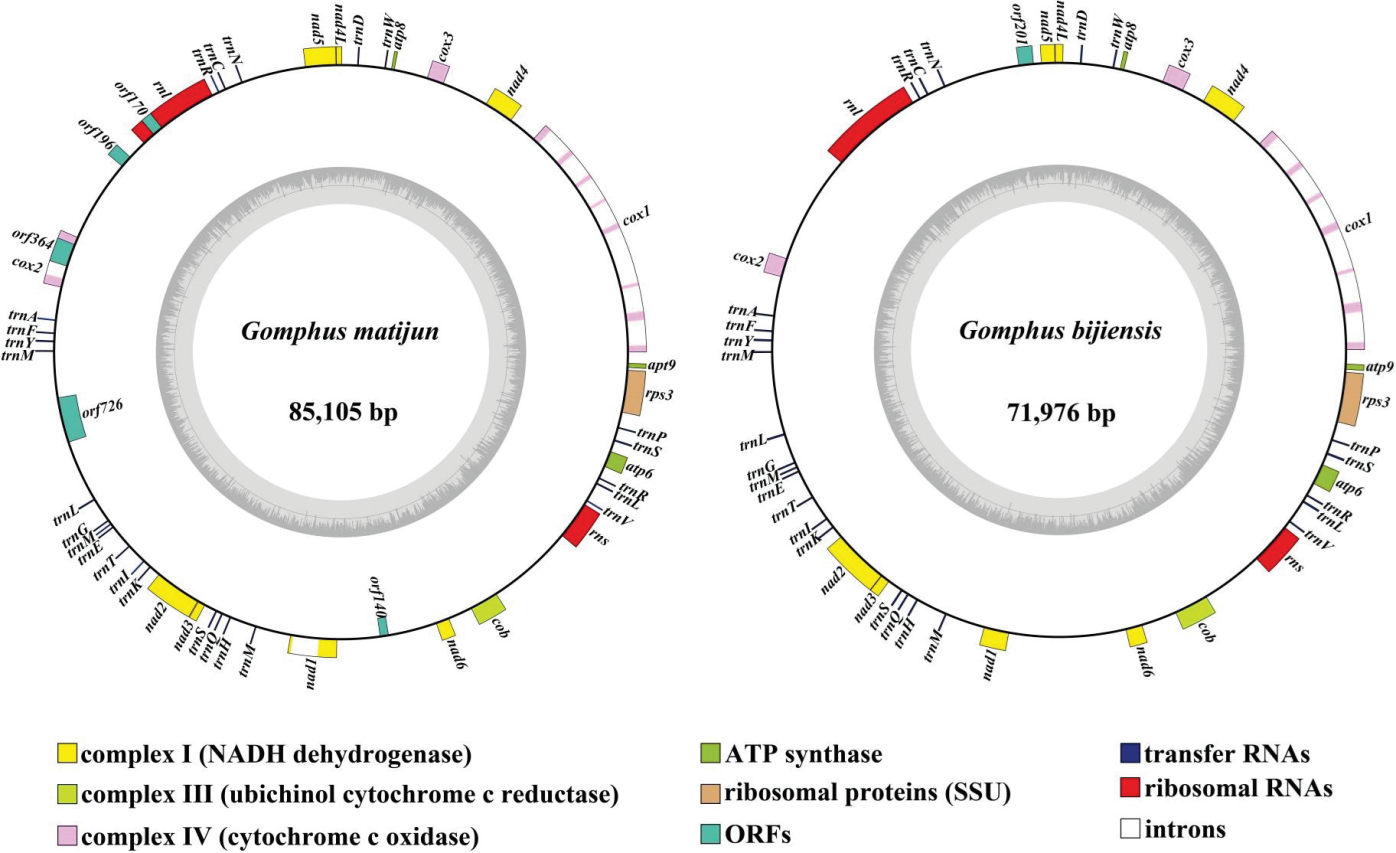
Depictions of the circular organization of the two *Gomphus* mitochondrial genomes. Various genes are illustrated as coloured blocks.

The mitogenomes of *G. bijiensis* and *G. matijun* were successfully assembled, with total lengths of 85,105 bp and 71,976 bp, respectively (Fig. 4). The GC content of the mitogenomes was 24.8% for *G. bijiensis* and 24.7% for *G. matijun* (Suppl. material 1: table S2). Strand asymmetry analysis indicated that both mitogenomes exhibited negative AT skews and positive GC skews, consistent with the patterns observed in other fungal mitogenomes. Each mitogenome contained a complete set of protein-coding genes (PCGs), including *atp6*, *atp8*, *atp9*, *cob*, *cox1*, *cox2*, *cox3*, *nad1*, *nad2*, *nad3*, *nad4*, *nad4 L*, *nad5*, *nad6*, and *rps3* (Suppl. material 1: table S3).

In addition to the complete set of protein-coding genes, both mitogenomes possessed genes encoding small and large subunit ribosomal RNAs (*rns* and *rnl*), as well as a complete set of transfer RNA (tRNA) genes. The tRNA genes displayed a typical fungal arrangement and encoded the 25 tRNAs necessary for mitochondrial protein synthesis, each corresponding to a specific amino acid. The tRNAs displayed the typical cloverleaf secondary structure, with several anticipated to include modified nucleotides frequently observed in fungal mitochondrial genomes, thereby enhancing the efficacy of the mitochondrial translation machinery.

Interestingly, significant variations were observed in the length and composition of intergenic regions between the two species. These regions, comprising non-coding sequences interspersed between genes, exhibited a higher AT content compared to the coding regions and likely play a role in regulating mitochondrial gene expression. The larger mitogenome of *G. bijiensis* contained longer intergenic spacers, which may have contributed to its overall genome size. Further investigation into these non-coding regions could shed light on their potential functional significance in mitochondrial genome organization and evolution.

Additionally, the presence of introns within some protein-coding genes, particularly *cox1* and *nad5*, reflects the dynamic nature of fungal mitogenomes. These introns, which often encode homing endonucleases, are believed to contribute to the lateral transfer of genetic material and influence the evolution of mitochondrial genomes. The differing occurrence in *G. bijiensis* and *G. matijun* indicates divergent evolutionary histories for these species, likely influenced by unique ecological and environmental factors.

#### ﻿Codon usage bias in *G. bijiensis* and *G. matijun*

**Figure 5.**
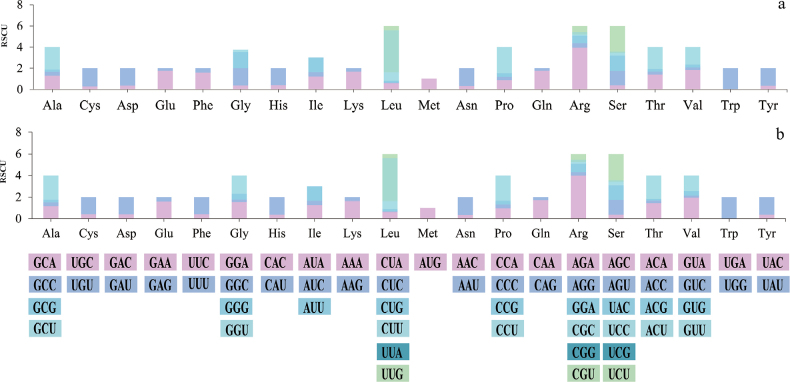
Relative synonymous codon usage (RSCU) in the mitochondrial genomes of two *Gomphus* species, presented as stacked column plots, a. *G. matijun*; b. *G. bijiensis*.

The relative synonymous codon usage (RSCU) weres analyzed for the mitochondrial genomes of *G. bijiensis* and *G. matijun* (Fig. 5). Both species exhibited distinct patterns of codon usage across the 20 amino acids. In *G. matijun*, the most frequently used codons included UUA for leucine (Leu) and AGA for serine (Ser), with RSCU values significantly higher than those of other synonymous codons, indicating a strong codon bias towards these codons. Similarly, codons CGA for arginine (Arg) and GGU for glycine (Gly) showed relatively higher usage, reflecting their preferential use in the mitochondrial genome. For *G. bijiensis*, UUA (Leu) again emerged as the most favored codon, displaying the highest RSCU value. Codons for serine (AGU), arginine (CGA), and glycine (GGU) also demonstrated high usage frequencies, aligning closely with the patterns observed in *G. matijun*. This suggests a conserved codon preference between the two species for certain amino acids, despite the differences in their overall mitochondrial genome sizes. Both species exhibited a pronounced preference for codons ending in A or U, consistent with their overall low GC content, as previously observed. The RSCU values across the codons suggest that specific codons are selectively preferred, which may be linked to the mitochondrial genome’s translational efficiency and its role in adaptive evolution.

#### ﻿Comparative genomic analysis

**Figure 6.**
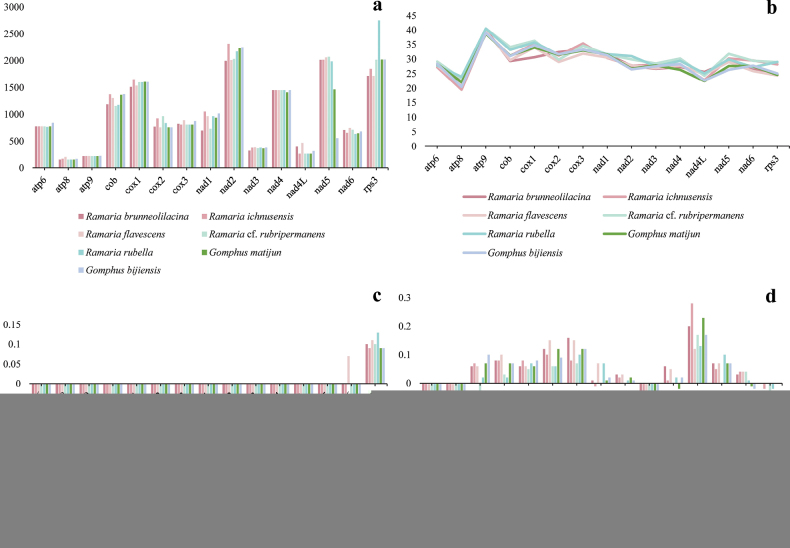
Differences in the length and base composition of 15 protein-coding genes (PCGs) across seven gomphoid mitochondrial genomes. a. PCG length variation; b. GC content across PCGs; c. AT skew; d. GC skew.

Genomic parameter analysis of seven gomphoid mitochondrial genomes, covering *Ramaria* and *Gomphus* species, revealed significant interspecific variations in key metrics (Fig. 6). The lengths of 15 protein-coding genes (PCGs) diverged remarkably among species, with the maximum observed length approaching 2,500 bp (e.g., in *G. matijun* at certain loci), likely reflecting structural differentiation and potential functional specialization related to metabolic or environmental adaptations. The GC content of PCGs exhibited a species-specific distribution range of ~10%–45%, with most genomes maintaining relative stability, but notable deviations were observed in some taxa, possibly associated with evolutionary adaptations such as enhanced DNA/RNA secondary structure stability or biased codon usage. The AT skew, quantifying A/T abundance bias (AT skew = (A - T)/(A + T)), varied from - 0.15 to 0.15, with positive values (adenine enrichment) in some *Ramaria* lineages and negative values (thymine bias) in partial *Gomphus* species, relating to strand-specific mutational pressures or gene expression constraints. Similarly, the GC skew (calculated as (G - C)/(G + C)), ranging from - 0.15 to 0.15, showed species-specific positive (guanine enrichment) or negative (cytosine enrichment) biases, driven by asymmetric DNA repair or functional constraints on coding sequences, potentially influencing lineage-specific evolutionary trajectories by modulating mutation rates and selective pressures.

#### ﻿Collinearity analysis

**Figure 7.**
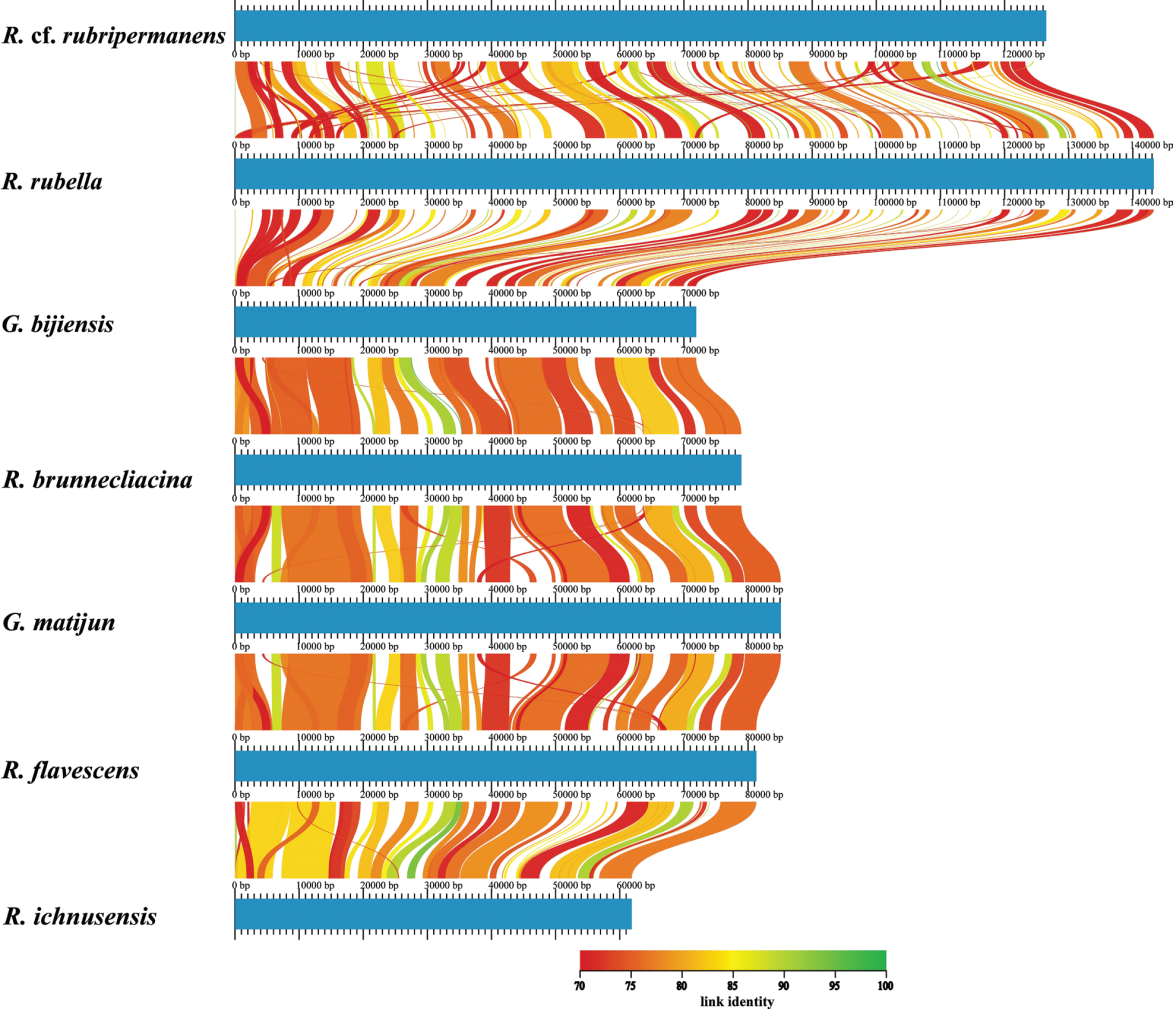
Genomic synteny analysis: unveiling evolutionary relationships among seven species; the darker the link colour, the closer the relationship between the homologous regions.

The collinearity analysis conducted across eight related species revealed distinct patterns of genomic conservation, as evidenced by the synteny plots comparing each pair’s genomes side by side (Fig. 7). Notably, there was a high degree of synteny observed between some pairs, such as *G. bijiensis* and *R. bruneilacinia*, where dense clusters of syntenic regions were presented throughout the length of the alignment, indicating strong genetic linkage and possibly closer evolutionary relationships. In contrast, other pairs demonstrated more sporadic syntenic connections, with significant portions lacking any notable linkage, suggesting greater genomic divergence over time or possible rearrangements post-speciation events. The comparison between *R. rubripiremans* Corner and *R. ichunensis* Zang showed fewer syntenic blocks, indicating a higher degree of genomic rearrangement.

#### ﻿Phylogenetic analysis

**Figure 8.**
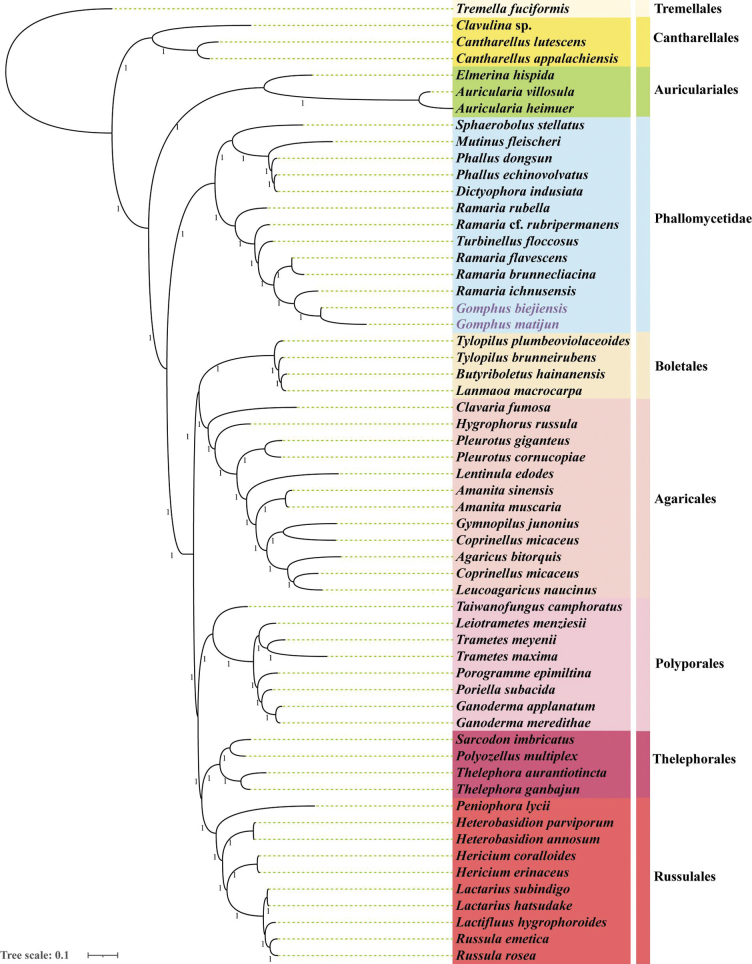
Phylogenetic analysis of 59 species (Suppl. material 1: table S4) using Bayesian inference (BI), based on 15 PCGs and 2 rRNA sequences. Species included in this study are marked with purple text.

In this study, we evaluated the phylogenetic status of 59 Basidiomycota species using a combined dataset of mitochondrial genes. A phylogenetic tree identical to and well-supported by both BI and ML methods was constructed (Suppl. material 2). The phylogenetic tree (Fig. 8) illustrates the evolutionary relationships among the studied species, with a particular focus on *G. bijiensis* and *G. matijun*, highlighted in purple. The analysis was conducted using Maximum Likelihood, and bootstrap values were calculated to assess the reliability of the inferred relationships. *G. bijiensi* and *G. matijun* form a distinct clade with high bootstrap support, indicating a close evolutionary relationship. This clade is nested within a larger group that includes several other species of the genus *Gomphus*, suggesting a common ancestor. The phylogenetic tree also reveals that *G. bijiensis* and *G. matijun* share a more recent common ancestor with each other than with any other species in the analysis. Other significant clades include the *Ramaria* clade, with species such as *R. rubella* and *R. flavescens* forming a well-supported clade, and the Trametes clade, comprising species like *T. maxima* (Berk.) Giachini, Hosaka & Trappe and *T. meyenii* (Lév.) Giachini, Hosaka & Trappe, which are grouped together, indicating a strong evolutionary link. *Ganoderma applanatum* (Pers.) Pat.and *Ganoderma meredithae* Adask. & Gilb. also exhibit close evolutionary ties, suggesting a recent divergence from a common ancestor.

#### ﻿Time divergence analysis

**Figure 9.**
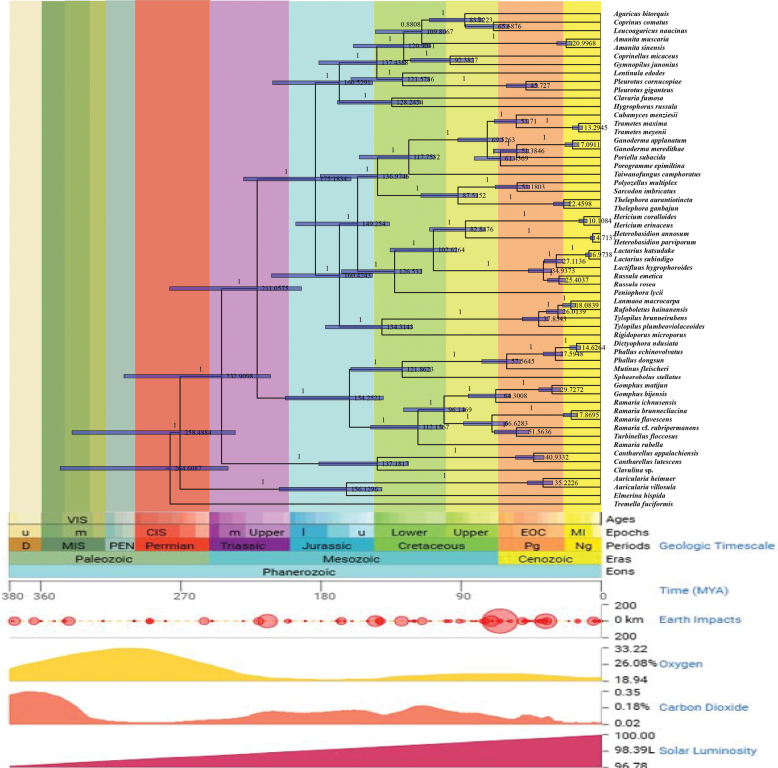
Phylogenetic analysis of basidiomycetes based on temporal analysis.

The phylogenetic analysis and timeline reveal that macrofungi with agaricoid (Fig. 9), coral-shaped, and spike-shaped morphologies have evolved independently across multiple lineages. Agarics, such as *Agaricus bisporus* (J.E. Lange) Imbach and *Amanita muscaria* (L.) Lam., emerged more recently during the Cenozoic era, likely in response to changing ecological conditions. Coral-shaped fungi, represented by *Clavaria fumosa* Pers. and *R. rubella* (Schaeff.) Quél., have a broader evolutionary timeframe, with origins dating back to the Jurassic period, suggesting early adaptation to diverse environments. Spike-shaped fungi, including *Hericium erinaceus* (Bull.) Pers. and *Auricularia heimuer* J.Z. Ying, exhibit a wide temporal range, indicating that this morphology has been a successful strategy across various ecological contexts.

## ﻿Discussion

Macrofungi mitochondrial genomes represent a valuable source of information for understanding the evolutionary history, adaptation strategies, and taxonomic relationships of diverse lineages. Patterns of nucleotide composition, codon usage bias, gene content, and genome organization often reflect both conserved functional constraints and lineage-specific evolutionary pressures. Comparative mitochondrial studies have demonstrated that closely related species can share highly conserved genomic features while simultaneously exhibiting distinct adaptations linked to ecology, metabolism, or life history strategies (Wang et al. 2025c). In this context, analyses of newly sequenced mitogenomes not only provide insights into species-level taxonomy but also contribute to broader understanding of fungal genome evolution, phylogeny, and ecological diversification.

The analyses of codon usage biases in the mitogenomes of *G. bijiensis* and *G. matijun* reveal several important insights into the genetic architecture and evolutionary dynamics of these species. The observed codon preferences, particularly the significant bias towards UUA (Leu) and AGA (Ser) codons, reflect a selective pressure towards optimizing translational efficiency in the mitochondrial environment. This pattern aligns with findings in other fungal species (Tao et al. 2024), where codon usage bias is commonly influenced by the combined effects of mutational pressures and natural selection. A strong preference for codons ending in A or U in both species is indicative of a low GC content, a common feature in many mitochondrial genomes. This bias may influence the structural stability of the mitochondrial RNA and could be an adaptation to the specific metabolic demands of these organisms. The conserved usage patterns of certain codons between *G. bijiensis* and *G. matijun* further suggest that these codons may play a crucial role in the functional optimization of mitochondrial genes, particularly those involved in critical cellular processes such as oxidative phosphorylation. Overall, the codon usage bias observed in these two *Gomphus* species underscores the importance of nucleotide composition and codon preference in shaping mitochondrial genome evolution. These findings provide a foundation for further comparative studies across other related species, which may shed light on the evolutionary pressures driving codon usage and genome organization in fungal mitochondria.

The observed variations in genomic parameters provide valuable insights into the evolutionary dynamics and genomic architecture of the studied species. The significant differences in PCG lengths among the species may reflect diverse evolutionary pressures and functional requirements. Longer PCGs could be associated with more complex protein functions or regulatory mechanisms, while shorter PCGs might indicate streamlined genetic coding for essential functions. The relatively stable GC content within each species implies that GC content is a conserved feature, potentially associated with genome stability and replication fidelity. Species exhibiting elevated GC content may have developed particular adaptations that provide advantages in their surroundings, such as enhanced thermal stability of DNA. The variations in AT and GC skew across the species highlight the asymmetric nature of nucleotide composition in their genomes. Positive AT or GC skews could indicate strand-specific mutational biases or selective pressures favoring certain nucleotide compositions. These skews may also be related to replication and transcription processes, influencing gene expression and genome organization. Overall, these findings underscore the complexity of genomic evolution and the diverse strategies employed by different species to adapt to their environments. Further investigation into the functional implications of these genomic features could provide deeper insights into the molecular mechanisms driving evolutionary change.

The patterns identified in our collinearity research offer significant insights into the evolutionary dynamics of these species’ groups. The extensive synteny shared by certain pairs, such as *G. bijiensis* and *R. bruneilacinia* Schild & T. Ohenoja, could imply recent common ancestry or strong selective pressures maintaining gene order across these genomes. This high degree of conservation may be indicative of essential biological functions that have been preserved over evolutionary time. Conversely, the scattered nature of syntenic blocks in other comparisons, such as between *R. rubripiremans* Marr & D.E. Stuntz and *R. ichunensis* Y.C. Dai & F. Wu, may reflect either ancient divergence events leading to significant genome reshuffling or adaptive responses to disparate ecological niches driving genome evolution independently within each lineage. These findings underscore the complexity inherent in genome evolution processes, while also highlighting potential avenues for further investigation. They pinpoint specific genes within highly conserved regions that may be critical for shared biological functions among these species.

The time divergence analysis provides significant insights into the evolutionary history of the studied species. The high bootstrap support for various clades suggests robust evolutionary relationships, consistent with previous studies indicating close genetic and morphological similarities among these species. *G. bijiensis* and *G. matijun* form a distinct clade, indicating a close evolutionary relationship. This clade’s placement within the larger *Gomphus* group suggests that these species have retained several ancestral traits while also developing unique characteristics, potentially driven by specific ecological niches or adaptive pressures unique to their environments. The clear delineation of various clades, including the *Gomphus* and *Ramaria* clades, has important taxonomic implications, supporting the classification of these species within their respective genera and highlighting the need for further taxonomic revisions to accurately reflect their evolutionary relationships. From a conservation perspective, understanding the evolutionary relationships and genetic diversity within these genera is crucial. Conservation strategies should consider the unique evolutionary history of species like *G. bijiensis* and *G. matijun* to ensure the preservation of their genetic diversity and ecological roles.

The morphological analysis of *G. bijiensis* spores, conducted under both optical and electron microscopes, has provided critical insights into its taxonomic classification within the *Gomphus* genus. The ellipsoidal to fusiform shape of the spores and their smooth surface, observed under optical microscopy, are characteristic of the genus. However, the distinct size range and surface microstructure identified through SEM analysis set *G. bijiensis* apart from closely related species. The SEM imagery, which revealed a finely textured surface with intricate ridges, suggests a potential adaptation to its ecological niche, possibly aiding in spore dispersal or environmental adherence.

The morphological features of the fresh basidiocarp also contribute to the identification of this new species. The robust, fleshy structure and distinctive cap-stipe differentiation, combined with the pigmentation patterns, suggest that *G. bijiensis* occupies a unique position within the genus. The observed characteristics suggest a potential adaptation to specific environmental conditions, which may involve interactions with soil microorganisms or symbiotic relationships with surrounding flora. These morphological distinctions provide a strong foundation for classifying *G. bijiensis* as a novel species and open up avenues for further ecological and phylogenetic studies to understand its role in its native habitat.

The discovery and characterization of *G. bijiensis* adds to the biodiversity of the *Gomphus* genus and highlights the importance of microscopic analysis in fungal taxonomy. Future research should aim to explore the ecological roles and potential applications of *G. bijiensis*, including its interactions within its ecosystem and possible contributions to biotechnological fields.

The results indicate that the morphological diversity in large fungi is largely driven by convergent evolution, with similar forms evolving independently in response to similar ecological pressures. The umbrella shape likely evolved during the Cenozoic to enhance spore dispersal in forested environments, while the coral shape, with its much older origins, suggests an adaptation to humid or aquatic ecosystems. Spike-shaped fungi exhibit broad evolutionary success across various periods, underscoring the adaptive advantages of this morphology. Overall, the study highlights the importance of morphological adaptations in the evolutionary diversification of fungi and their ecological success in diverse niches.

## ﻿Conclusions

The complete mitogenomes of *G. bijiensis* and *G. matijun* were successfully assembled, measuring 85,105 bp and 71,976 bp, respectively. The GC content was 24.7% for *G. matijun* and 24.8% for *G. bijiensis*. Strand asymmetry analysis revealed negative AT skews and positive GC skews in both mitogenomes, consistent with patterns in other fungal mitogenomes. Each contained a complete set of protein-coding genes (PCGs), including *atp6*, *atp8*, *atp9*, *cob*, *cox1*, *cox2*, *cox3*, *nad1*, *nad2*, *nad3*, *nad4*, *nad4L*, *nad5*, *nad6*, and *rps3*. Analysis of genomic parameters across eight species demonstrated significant variations, with PCG lengths reaching up to 2,500 base pairs, indicating differences in gene structure and function. The GC content of PCGs ranged from 10% to 45%, with most exhibiting stability, while some showed higher GC content linked to evolutionary adaptations. The AT skew varied between -0.15 and 0.15, revealing species-specific abundances of adenine and thymine, while the GC skew showed similar ranges, indicating varied guanine and cytosine content. Divergence time analyses suggest that morphological diversity in macrofungi, including *Gomphus* species, has been shaped by convergent evolution. The phylogenetic tree places *G. bijiensis* and *G. matijun* in a distinct clade, indicating a close evolutionary relationship characterized by both the retention of ancestral traits and the emergence of unique adaptations. This study describes a new species and provides the first comprehensive mitochondrial genomic data for *Gomphus*, significantly advancing our understanding of its evolutionary relationships and divergence timelines. This, in turn, enhances *Gomphus* taxonomy and lays a foundation for future research on the evolutionary dynamics within the Gomphaceae family.

## Supplementary Material

XML Treatment for Gomphus bijiensis

XML Treatment for Gomphus matijun

## ﻿Additional information

### Use of AI

No use of AI was reported.

### Author ORCIDs

Qirui Li https://orcid.org/0000-0001-8735-2890

### Data availability

All data generated or analyzed during this study are included in this published article and its additional files. The complete mitogenomes of *G. bijiensis* and *G. matijun* were deposited in the GenBank database under the accession numbers PX373692 and PX373691, respectively. The ITS sequence of *G. bijiensis* is available in GenBank under accession number PV661541, and the LSU sequence is deposited under PV661542. The new species *G. bijiensis* is registered in the Fungal Name database with the registration number FN572719. All data are publicly accessible and can be retrieved through the provided accession numbers.

## Supplementary materials

Supplementary material 1 Additional file Data type xlsx https://binary.pensoft.net/file/1465160This dataset is made available under the Open Database License (http://opendatacommons.org/licenses/odbl/1.0/). The Open Database License (ODbL) is a license agreement intended to allow users to freely share, modify, and use this Dataset while maintaining this same freedom for others, provided that the original source and author(s) are credited.Author: Zhongyao Guo

Supplementary material 2 Supplementary charts Data type pdf https://binary.pensoft.net/file/1465161This dataset is made available under the Open Database License (http://opendatacommons.org/licenses/odbl/1.0/). The Open Database License (ODbL) is a license agreement intended to allow users to freely share, modify, and use this Dataset while maintaining this same freedom for others, provided that the original source and author(s) are credited.Author: Zhongyao Guo
