# Supplementary material for: ﻿Phylogeny and comparative analysis of mitochondrial genomes of Gomphus spp. Pers. (Basidiomycota, Agaricomycetes), with descriptions of G. matijun J.W. Liu & F.Q. Yu and G. bijiensis sp. nov
Source: MycoKeys. 2025 Nov 13;124:357–81. doi: 10.3897/mycokeys.124.158670 (PMC12635650; doi:10.3897/mycokeys.124.158670)
Supplement: Supplementary material 2 — Supplementary charts [file mycokeys-124-357-s002.pdf]

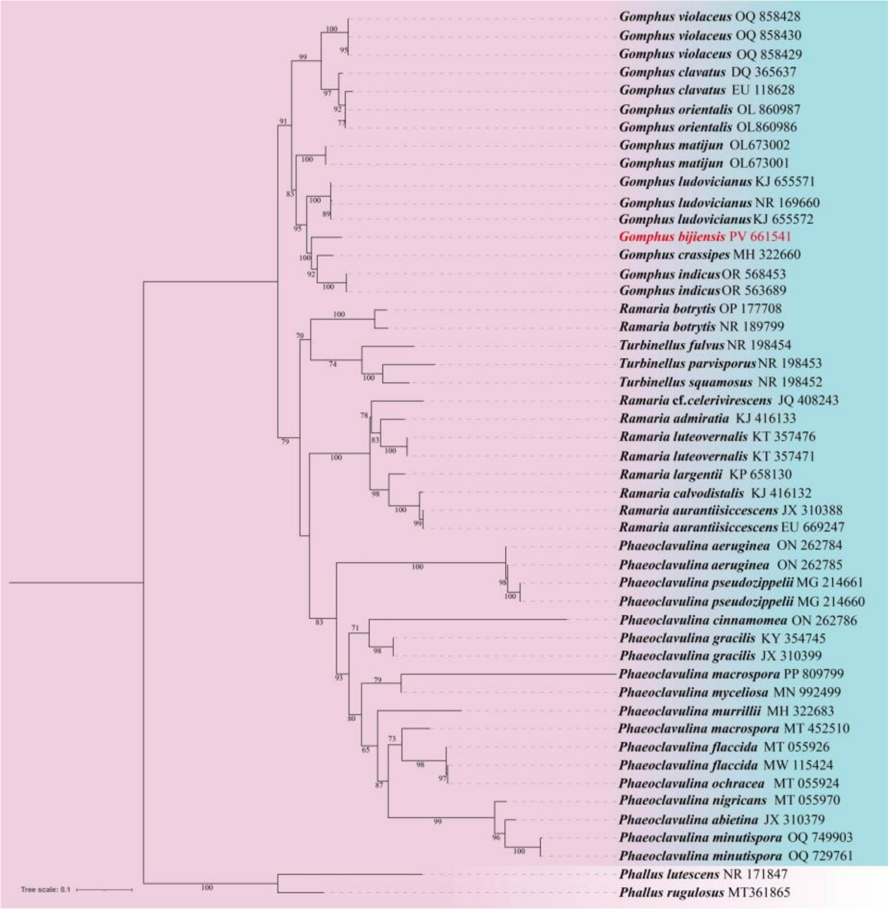

Maximum likelihood (ML) phylogeny of 49 fungal species based on ITS sequences

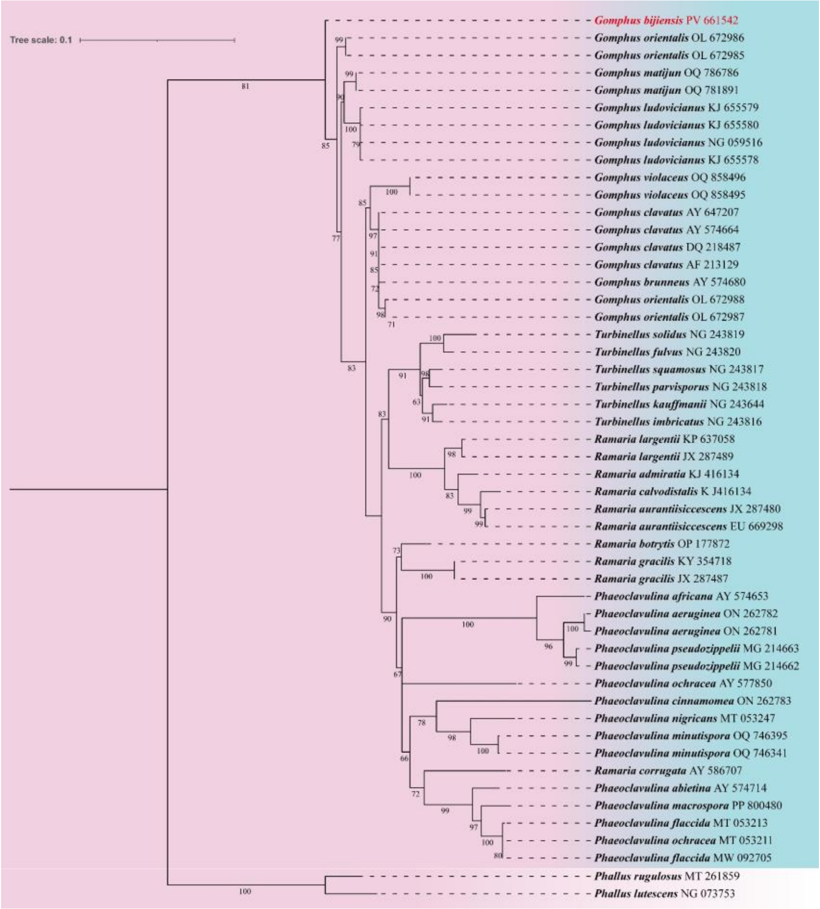

Maximum likelihood (ML) phylogeny of 51 fungal species based on LSU sequences

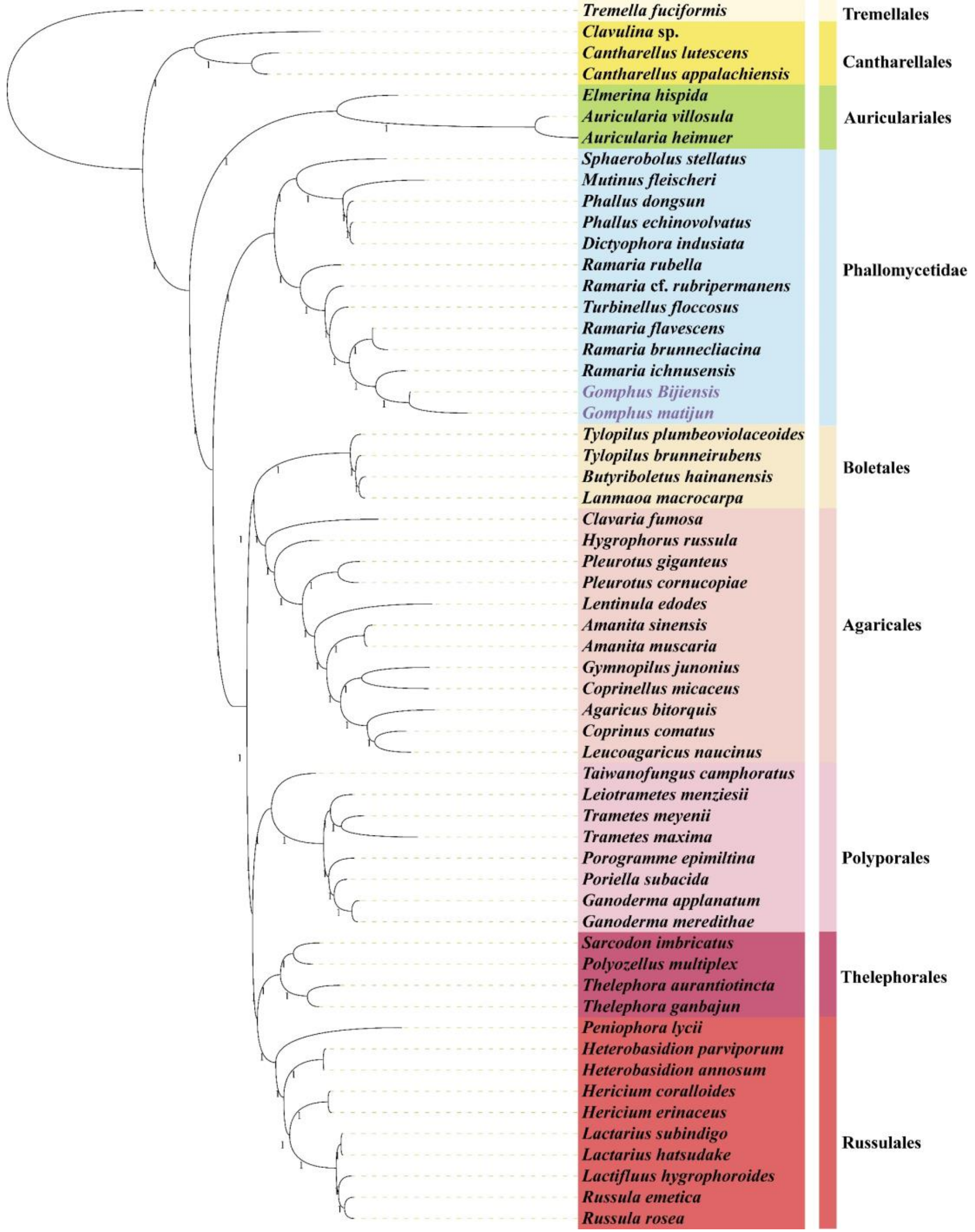

Phylogenetic analysis of 59 Basidiomycota species using Bayesian inference (BI), based on comprising the first and second codon positions of these 15 PCGs and 2rRNA sequences.

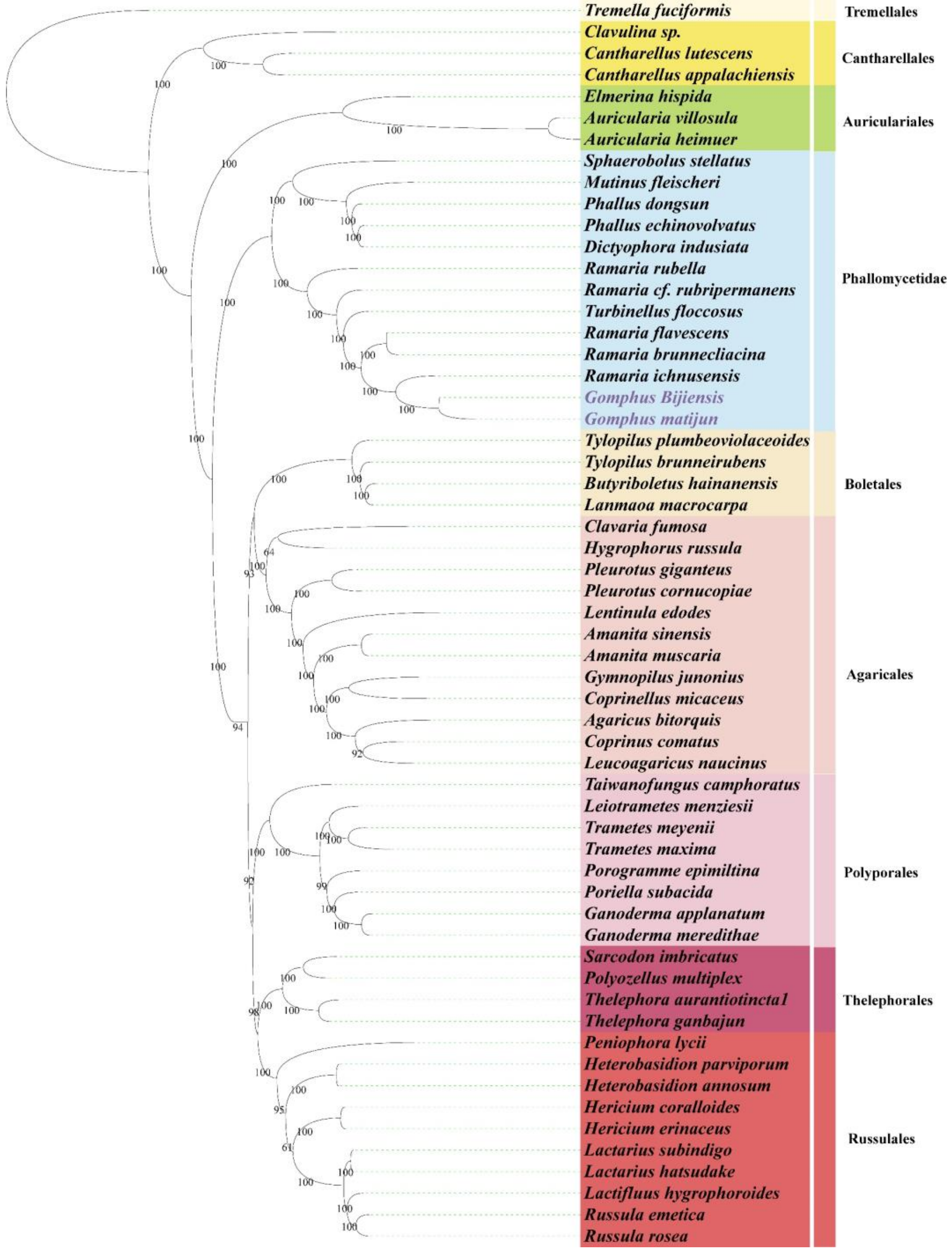

Phylogenetic analysis of 59 Basidiomycota species using maximum likelihood (ML), based on comprising the first and second codon positions of these 15 PCGs and 2rRNA sequences.

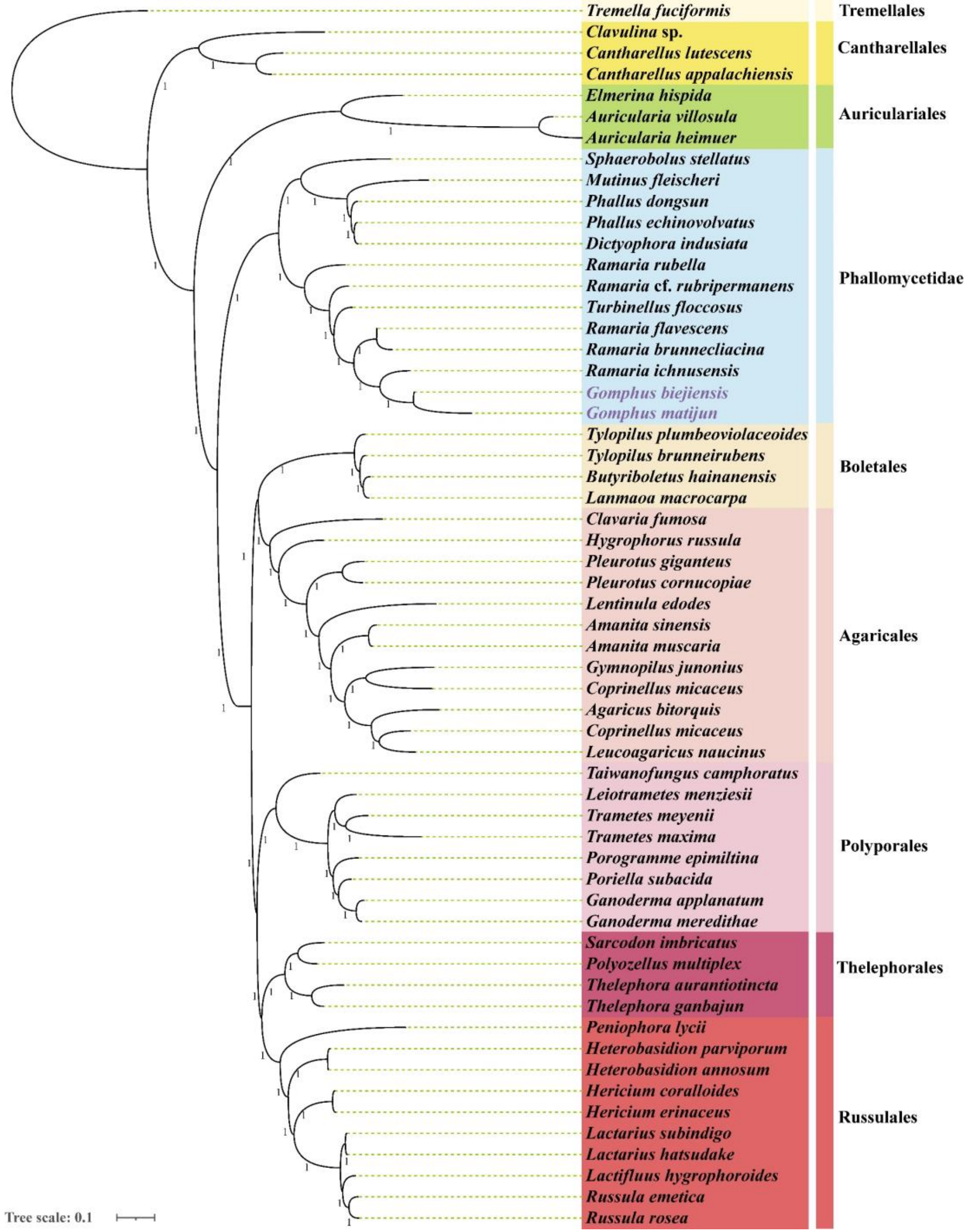

Phylogenetic analysis of 59 Basidiomycota species using Bayesian inference (BI), based on 15 PCGs and 2rRNA sequences.

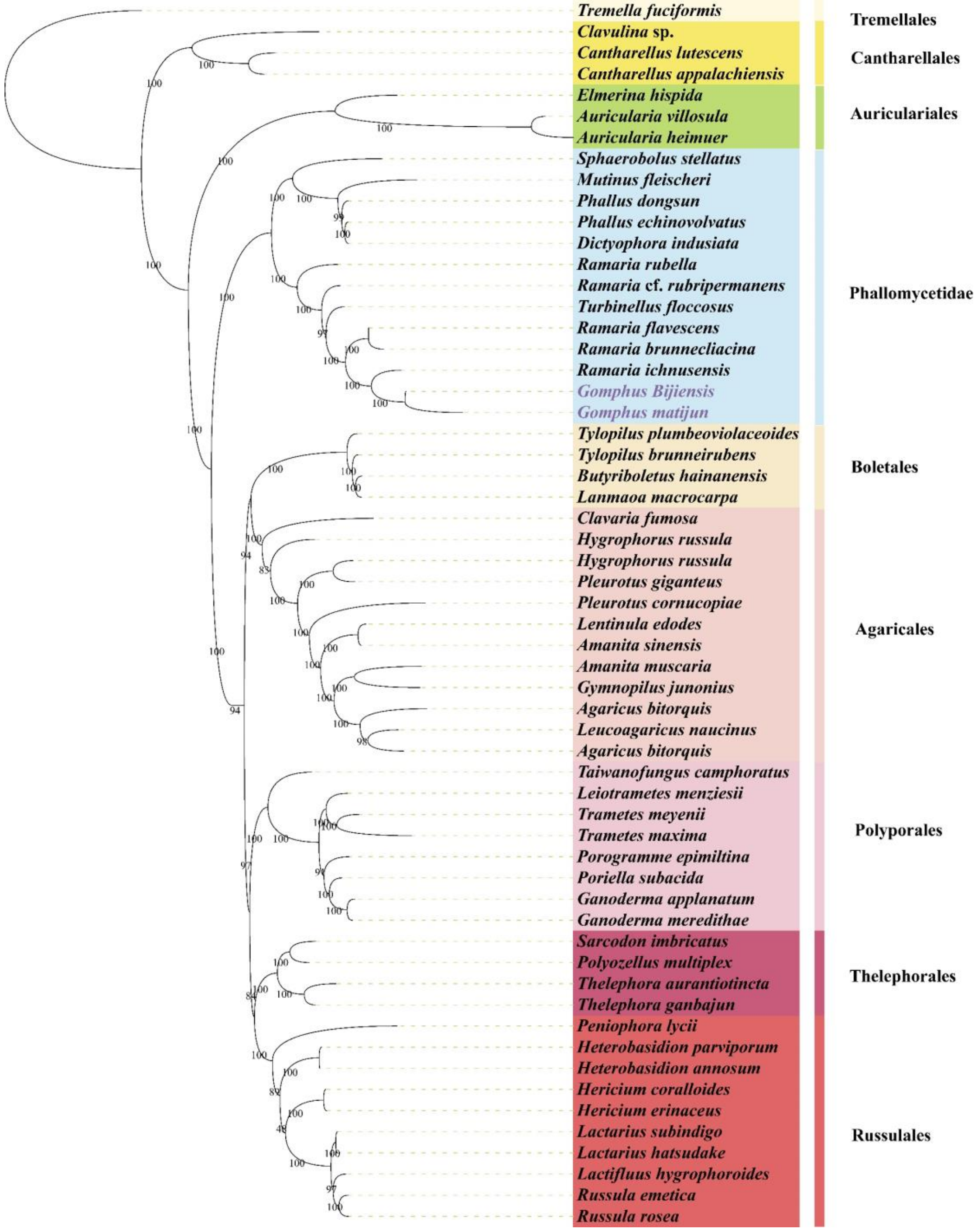

Phylogenetic analysis of 59 Basidiomycota species using maximum likelihood (ML), based on 15 PCGs and 2rRNA sequences.
